# Assessment of the health status and health service perceptions of international migrants coming to Guangzhou, China, from high-, middle- and low-income countries

**DOI:** 10.1186/s12992-019-0449-y

**Published:** 2019-01-25

**Authors:** Remina Maimaitijiang, Qiangsheng He, Yanan Wu, Jennifer Z. H. Bouey, Ahoua Koné, Yucheng Liang, Chun Hao, Jiong Tu, Jing Gu, Yuantao Hao

**Affiliations:** 1 Department of Medical Statistics and Epidemiology, School of Public Health, Sun Yat-sen University, School of Public Health, No. 74 Zhongshan Road 2, Guangzhou, 510080 Guangdong People’s Republic of China; 2 Department of International Health, Georgetown University, St. Mary’ s Hall, 3700 Reservoir Road, N.W., Washington D.C., 20057 USA; 3 Department of Global Health, University of Washington, Seattle, WA and Health Alliance International, 1107 NE 45th Street, Suite 350, Seattle, WA 98105 USA; 4 Department of Sociology and Center for Social Survey, Sun Yat-sen University, No. 135 Xingang Xi Road, Guangzhou, 510275 People’s Republic of China; 5 Sun Yat-sen Global Health Institute, Institute of State Governance, Sun Yat-sen University, No. 135 Xingang Xi Road, Guangzhou, 510275 People’s Republic of China

**Keywords:** International migrants, Health status, Perceptions of health services, China

## Abstract

**Background:**

China, which used to be an export country for migrants, has become a new destination for international migrants due to its rapid economic growth. However, little empirical data is available on the health status of and health service access barriers faced by these international migrants.

**Methods:**

Foreigners who visited the Guangzhou Municipal Exit-Entry Administration Office to extend their visas were invited to participate in the study. Quantitative data were collected using electronic questionnaire in 13 languages. The participants were characterised by the income level of their country of origin (high-, middle- and low-income countries (HICs, MICs and LICs, respectively)), and the key factors associated with their health status, medical insurance coverage and perceptions of health services in China were examined.

**Results:**

Overall, 1146 participants from 119 countries participated in the study, 57.1, 25.1 and 17.8% of whom were from MICs, HICs and LICs, respectively. Over one fifth of the participants experienced health problems while staying in China, and about half had no health insurance. Although the participants from HICs were more likely than those from MICs and LICs to have medical insurance, they were also more likely to have health problems. Furthermore, 43.0, 45.0 and 12.0% of the participants thought that the health services in China were good, fair and poor, respectively. Among the participants, those from HICs were less likely to have positive feedback.

**Conclusions:**

Our study is the first to report a quantitative survey of the health status, health insurance coverage, and health service perceptions of a diverse and surging population of international migrants in China. The findings call for more in-depth studies on the challenges presented by the increasing global migration to the health system.

## Background

Over the past few decades, migration has become a key feature of our increasingly interconnected and globalised world. According to the United Nations, the number of international migrants worldwide has increased from 76 million people in 1960 to 232 million people in 2013 [1]. International migration can be divided into four types based on the country of origin and host country. Although most attention is on the movement of people from developing countries to developed countries, the second-largest flow of people is between developing countries, so called South-to-South migration [2]. According to the 2017 International Migrant Report, Asia now receives more international migrants (80 million) than Europe (78 million) or North America (58 million) [3].

In recent years, China has become a new destination for international migration because of its rapid economic development [1]. In 2013, it was estimated that about 848,500 foreigners lived in China, with an average annual growth rate of 3.9% [1]. As the paragon of China’s economic reform and a major trading port of southern China, the city of Guangzhou in Guangdong province has attracted a large number of international migrants from both developed and developing countries [4]. In 2017, the number of foreigners living in Guangzhou was estimated to be 88,000 [5]. This large-scale inflow of international visitors to Guangzhou has not only boosted the local economic prosperity of the city and reformed the social and cultural landscape but also created challenges, including health-related issues [6].

Although it is often reported that the majority of international migrants are relatively young and healthy, this assertion should be reassessed given a significant increasing trend in chronic diseases among young and middle-aged populations worldwide, including in developing countries [7], along with a relatively high prevalence of infectious diseases (e.g. HIV and neglected tropical diseases) among international migrants from certain regions [8]. Furthermore, because international migrants often encounter challenges such as language barriers, economic difficulties, and lack of access to health services in the process of adapting to the foreign environment [9–12] in addition to cultural conflicts and racial discrimination, they tend to become more vulnerable than the locals to other mental and physical ailments [13, 14].

A study in the US reported that compared with the local population, international migrants have lower mortality rates and are less likely to suffer from circulatory diseases, obesity and some cancers [15]. However, one systematic review from Europe reported that international migrants are more likely than non-migrants to self-rate their health status as poor [16]. Thus, the findings from literature are inconsistent with regard to study populations and health outcomes [17]. In addition, migration-related factors, such as language barriers, economic status, cultural differences and legal status, have been reported to be associated with the health status of and health service utilisation among international migrants [18–21].

Most of the current research on international migrants has been conducted in developed countries, and therefore, the findings are not applicable to South-to-South migrants [22]. Furthermore, in most China-related studies on international migrants, China has been presented as an exporting country. However, in recent years, as China is receiving increasing number of foreigners [1], it is important to understand the migrants’ demographic characteristics, health status and health-seeking behaviour to build a resilient health system. Research concerning such issues in China is very limited [7]. In addition, almost all of the few studies available on South-to-South migrants to China are qualitative and specific to African migrants and their low utilisation of community health services [23] or their dissatisfaction with access to health care and health services [24–26].

There is a lack of quantitative evidence on the health status of international migrants in China and their access to health services. This is critical given that the country of origin is considered as an important determinant in explaining the differences in the health status of and health service utilisation among migrant population [27, 28]. To help fill this gap, the present study aimed to quantitatively assess the key demographic and health service access characteristics of international migrants to China by the income level of their country of origin [29]. Moreover, it is one of the few studies focusing on the characteristics and health-related perceptions of international migrants coming from high-, middle- and low-income countries (HICs, MICs and LICs, respectively) to a developing country. We hope that this study will initiate a national debate on the development of policies for international migrants and prepare the health system for the challenges presented by an increasingly mobile global population.

## Methods

### Participants and setting

Similar to most studies on migrants and hidden populations, there is no available sampling frame for international migrants in Guangzhou. The participants in this study were adult foreign visitors (age: 18 years or above) who visited the Guangzhou Municipal Exit-Entry Administration Office to extend their visa during January 2016. China grants permanent residency to a small number of foreigners (0.06%) [30] compared with other prominent countries that grant it to about 10% of foreigners. Therefore, most foreign visitors in Guangzhou who desire a long-term residence must go through a lengthy visa extension process [31] at the Guangzhou Municipal Exit-Entry Administration Office.

### Procedure

While the foreign migrants were waiting in the Municipal Exit-Entry Administration Office for a visa extension, they were approached by trained research assistants from Sun Yat-sen University and invited to participate in the survey. Eligible participants were briefed about the study aims, assured of data confidentiality and requested to voluntarily participate by signing consent forms provided in 13 languages, including Chinese, English, French, Portuguese, Spanish, Arabic and Swahili. Individuals who agreed to participate in the study completed an electronic questionnaire on electronic tablets with help from the research assistants. The survey was also available in 13 languages and did not contain any identifiable information, such as name or passport number. After completion of the survey, the participants were provided a small gift (postcards) for participation. Non-response accounted for about 30% of the individuals who were invited to participate in the survey. Eventually, 1162 participants who responded were recruited, 1146 (98.6%) of whom completed the survey. The study was approved by the Ethics Committee of the Faculty of Sociology and Anthropology of Sun Yat-sen University.

### Measures

Data were collected from the recruited participants on their socio-demographic characteristics including gender, age, education level, current marital status and job status in China as well as migration-related characteristics including their visa type, the number of years since their first visit to China, the total number of visits they made and the number of months they have been staying in China. The participants’ social integration in China was assessed by whether they had a Chinese name (yes or no), whether they had an education experience in China (yes or no) and their proficiency in Mandarin and English (not speaking the language at all, poor, sufficient for daily life or excellent). The participants’ social network in China was measured by the number of their Chinese acquaintances and fellow townsmen they knew in Guangzhou as well as their experience of participating in hometown associations or the Chamber of Commerce in Guangzhou.

In addition to the questionnaire and the above assessments, each participant was asked five questions regarding their health-related status. Three of these questions enquired about their perceived health status: how they perceived their health status (poor, fair, good or very good); the change in their health status since they came to China (became less healthy, no change or this is their first time to China, or became healthier); and whether they ever had health problems in China (no, yes or this is their first time to China). The fourth question enquired whether they had medical insurance while staying in China (yes or no), and the fifth question enquired about their overall assessment of China’s health services (poor, fair or good).

Using the World Bank classification, we divided the participants into three groups: those originating from LICs, those from MICs and those from HICs.

### Statistical analyses

The differences in five groups of variables (socio-demographic variables, migration-related variables, social integration, social network in China and health-related variables) were compared between the three country groups (LIC, MIC and HIC groups). Chi-square test, one-way analysis of variance and non-parametric tests were used for data analysis. Bonferroni adjustment of the significance level was applied for multiple comparisons.

Logistic regression models were used to identify variables associated with the five health-related outcome variables (perceived good health status, became healthier since they came to China, ever had health problems in China, had medical insurance in China and perceived health services in China as good). To examine the independent effects of the income level of the countries of origin on various outcomes, adjusted logistic regression models were applied after adjusting for factors with *p* < 0.1 in previous univariate analyses. All statistical analyses were performed using the SPSS software version 21.0.

## Results

### Socio-demographic and migration-related characteristics

The 1146 participants were from 119 countries; 57.1% (*n* = 654) from MICs, 25.1% (*n* = 288) from HICs and 17.8% (*n* = 204) from LICs (Table 1). The top three countries in the MIC group were India, Yemen and Russia; in the HIC group were Korea, Japan America; and in the LIC group were Congo, Tanzania and Ethiopia. Furthermore, 74.8% of the participants were male, and the average age of the participants was 31.3 years (SD = 9.7 years). Participants in the HIC group were older (36.1 years) those in the other two groups (LIC group: 29.2 years; MIC group: 29.8 years; *p* < 0.001). Most participants had received college level or higher education (81.0%), especially those in the HIC group (85.4%). Approximately half (52.4%) of the participants were single, and 60.3% had a job in China.

**Table 1:** Participants’ socio-demographic and migration-related characteristics by income levels of countries of origin

	LICs	MICs	HICs	All	*P* value
	204 (17.8%)	654 (57.1%)	288 (25.1%)	1146	
Socio-demographic characteristics					
Gender					0.021^a^
Male	166 (81.4)	471 (72.0)	220 (76.4)	857 (74.8)	
Female	38 (18.6)	183 (28.0)	68 (23.6)	289 (25.2)	
Age (mean ± SD)	29.2 ± 7.5	29.8 ± 8.8	36.1 ± 11.4	31.3 ± 9.7	< 0.001^bc^
Education level					0.030^bc^
Junior high or below	13 (6.4)	41 (6.3)	7 (2.4)	61 (5.3)	
Senior high school	31 (15.3)	90 (13.8)	35 (12.2)	156 (13.6)	
College or above	158 (78.2)	522 (79.9)	246 (85.4)	926 (81.0)	
Current marital status					0.012^c^
Married	68 (33.3)	265 (40.6)	125 (43.4)	458 (40.0)	
Cohabitation	15 (7.4)	41 (6.3)	31 (10.8)	87 (7.6)	
Single	121 (59.3)	347 (53.1)	132 (45.8)	600 (52.4)	
Currently has a job in China					< 0.001^bc^
No	101 (49.5)	271 (41.4)	83 (28.8)	455 (39.7)	
Yes	103 (50.5)	383 (58.6)	205 (71.2)	691 (60.3)	
Migration-related characteristics					
Visa type					< 0.001^abc^
Tourist visa	20 (9.8)	100 (15.4)	48 (16.9)	168 (14.7)	
Business visa	58 (28.4)	158 (24.3)	54 (19.0)	270 (23.7)	
Work visa	17 (8.3)	161 (24.7)	98 (34.5)	276 (24.2)	
Student visa	96 (47.1)	167 (25.7)	38 (13.4)	301 (26.4)	
Others	13 (6.4)	65 (10.0)	46 (16.2)	124 (10.9)	
Number of years since the first visit to China					< 0.001^abc^
> 10 years	16 (8.0)	110 (16.9)	68 (24.1)	194 (17.1)	
6–10 years	40 (20.1)	143 (21.9)	82 (29.1)	265 (23.4)	
< 6 years	143 (71.9)	399 (61.2)	132 (46.8)	674 (59.5)	
Number of visits to China					< 0.001^abc^
Once	77 (37.9)	162 (24.8)	48 (16.7)	287 (25.1)	
2–5 times	68 (33.5)	218 (33.4)	91 (31.6)	377 (33.0)	
6–10 times	24 (11.8)	84 (12.9)	38 (13.2)	146 (12.8)	
> 10 times	34 (16.7)	189 (28.9)	111 (38.5)	334 (29.2)	
Number of cumulative months of stay in China					< 0.001^ac^
0–6	97 (50.0)	440 (69.3)	180 (64.7)	717 (64.8)	
7–12	20 (10.3)	66 (10.4)	27 (9.7)	113 (10.2)	
13–60	52 (26.8)	77 (12.1)	41 (14.7)	170 (15.4)	
61≤	25 (12.9)	52 (8.2)	30 (10.8)	107 (9.7)	

^a^Differences between participants from LICs and those from MICs were statistically significant

^b^Differences between participants from MICs and those from HICs were statistically significant

^c^Differences between participants from LICs and those from HICs were statistically significant

About a quarter of the participants were holding either a business visa (23.7%, Table 1), a work visa (24.2%) or a student visa (26.4%). About one third of the participants (29.2%) had visited China more than 10 times, and 25.1% had spent more than 12 cumulative months in China. The HIC group visited China more frequently than the other two groups (*p* < 0.001), whereas the LIC group stayed in China longer than the other two groups (*p* < 0.001).

### Social integration and social network-related characteristics

About half of the participants (47.1%) had a Chinese name, and 41.5% took classes in educational institutes in China (Table 2). The percentage of participants who had education experience in China was 52.2, 42.2 and 32.4% for the LIC, MIC and HIC groups, respectively, and the differences between the three groups were significant (*p* < 0.001). About 40% of the participants spoke little Mandarin, whereas 19.5% did not speak the language at all. Most of the participants (56.1%) were fluent in English, and 16.5% spoke little or no English. No statistically significant differences were observed in language proficiency between the three groups.

**Table 2:** Participants’ social and health-related characteristics by income levels of countries of origin

	LICs	MICs	HICs	All	*P* value
	204 (17.8%)	654 (57.1%)	288 (25.1%)	1146	
Social integration in China					
Has a Chinese name					0.433
No	115 (56.9)	340 (52.3)	148 (51.4)	603 (52.9)	
Yes	87 (43.1)	310 (47.7)	140 (48.6)	537 (47.1)	
Has education experience in China					< 0.001^abc^
No	97 (47.8)	377 (57.8)	194 (67.6)	668 (58.5)	
Yes	106 (52.2)	275 (42.2)	93 (32.4)	474 (41.5)	
Proficiency in Mandarin					0.316
Know nothing	45 (22.8)	109 (18.0)	58 (20.3)	212 (19.5)	
Know little	82 (41.6)	236 (39.1)	114 (39.9)	432 (39.8)	
Fair	46 (23.4)	176 (29.1)	74 (25.9)	296 (27.2)	
Fluent	24 (12.2)	83 (13.7)	40 (14.0)	147 (13.5)	
Proficiency in English					0.094
Know nothing	6 (2.9)	20 (3.1)	4 (1.4)	30 (2.7)	
Know little	33 (16.2)	73 (11.4)	50 (17.7)	156 (13.8)	
Fair	61 (29.9)	191 (29.8)	58 (20.5)	310 (27.5)	
Fluent	104 (51.0)	358 (55.8)	171 (60.4)	633 (56.1)	
Social network in China					
Number of Chinese acquaintances in Guangzhou					0.107
0–10	95 (47.0)	232 (35.5)	113 (39.4)	440 (38.5)	
11–50	51 (25.2)	209 (32.0)	81 (28.2)	341 (29.8)	
51≤	56 (27.7)	213 (32.6)	93 (32.4)	362 (31.7)	
Number of fellow townsmen known in Guangzhou					< 0.001^bc^
0–10	94 (47.7)	302 (47.6)	186 (65.5)	582 (52.2)	
11–50	49 (24.9)	193 (30.4)	65 (22.9)	307 (27.5)	
51≤	54 (27.4)	140 (22.0)	33 (11.6)	227 (20.3)	
Participation in hometown associations/the Chamber of Commerce in Guangzhou					0.061
No	156 (77.2)	529 (82.1)	243 (85.6)	928 (82.1)	
Yes	46 (22.8)	115 (17.9)	41 (14.4)	202 (17.9)	
Health status and perception of health services					
Perceived health status					< 0.001^ab^
Poor or fair	13 (6.5)	65 (9.9)	41 (14.2)	119 (10.4)	
Good	64 (31.8)	274 (41.9)	117 (40.6)	455 (39.8)	
Very good	124 (61.7)	315 (48.2)	130 (45.1)	569 (49.8)	
Change in health status since coming to China					< 0.001^abc^
Became less healthy	15 (7.5)	115 (17.6)	57 (19.8)	187 (16.4)	
No change	135 (67.2)	412 (63.1)	204 (70.8)	751 (65.8)	
Became healthier	51 (25.4)	126 (19.3)	27 (9.4)	204 (17.9)	
Ever had health problems in China					< 0.001^ac^
No/first time to China	182 (89.2)	513 (78.9)	207 (71.9)	902 (79.0)	
Yes	22 (10.8)	137 (21.1)	81 (28.1)	240 (21.0)	
Had medical insurance while staying in China					< 0.001^bc^
No	117 (57.6)	331 (51.4)	108 (37.8)	556 (49.1)	
Yes	68 (33.5)	204 (31.7)	150 (52.4)	422 (37.2)	
Don’t know	18 (8.9)	109 (16.9)	28 (9.8)	155 (13.7)	
Overall perception of health services in China					< 0.001^bc^
Poor	14 (7.0)	57 (8.9)	63 (22.8)	134 (12.0)	
Fair (not good or bad)	78 (39.0)	278 (43.4)	146 (52.9)	502 (45.0)	
Good	108 (54.0)	305 (47.7)	67 (24.3)	480 (43.0)	

^a^Differences between participants from LICs and those from MICs were statistically significant

^b^Differences between participants from MICs and those from HICs were statistically significant

^c^Differences between participants from LICs and those from HICs were statistically significant

In terms of the social network of the participants in China, 61.5% knew more than 10 Chinese residents in Guangzhou, 47.8% knew more than 10 people from their home country in Guangzhou, and 17.9% participated in hometown associations/the Chamber of Commerce in Guangzhou. Participants from HICs knew less fellow townsmen in Guangzhou than the other two groups (*p* < 0.001).

### Health status and perception of health services in China

Most of the participants (89.6%) self-evaluated their health as good or very good (Table 2). Over one fifth of the participants (21.0%) reported having had health problems while staying in China. Participants from HICs (28.1%) were more likely to have had health problems in China as compared to the other two groups (LIC: 10.8%, MIC: 21.1%; *p* < 0.001). Most of the participants (65.8%) reported that their health status had not changed since they came to China (or this was their first time to China). However, participants from LICs (25.4%) were more likely than those from MICs (19.3%) and HICs (9.4%) to report an improved health status, whereas participants from HIC (19.8%) were more likely than those form LICs (7.5%) and MICs (17.6%) to report a deteriorated health status.

Only 37.2% of the participants reported that they had medical insurance during their stay in China. Most of those participants belonged to the HIC group (52.4%) compared with the other two groups (LIC: 33.5%, MIC: 31.7%). Furthermore, 43.0, 45.0 and 12.0% of all participants thought that the health services in China were good, fair and poor, respectively. Participants from HICs (22.8%) were more likely to have a negative perception of China’s health services than those from MICs (8.9%) and LICs (7.0%) (*p* < 0.001).

### Factors associated with participants’ health status, medical insurance, and perception of health services in China

After controlling for potential confounders (socio-demographic characteristics, migration-related characteristics, social integration and social network-related characteristics), participants from HICs were found to be less likely than those from LICs to have good health status in China (OR = 0.41, 95% CI = 0.21–0.80, *p* < 0.01, Table 3). Similarly, participants from HICs were less likely than those from LICs to become healthier since arriving in China (OR = 0.33, 95% CI = 0.19–0.56, *p* < 0.001). After controlling for potential confounders, the MIC (OR = 2.20, 95% CI = 1.25–3.85, *p* < 0.01) and HIC (OR = 3.53, 95% CI = 1.93–6.44, *p* < 0.001) groups were found to be more likely than the LIC group to have health problems in China. In addition, participants from HICs were more likely to have medical insurance in China (OR = 2.63, 95% CI = 1.67–4.15, *p* < 0.001) and were more likely to have negative perceptions of health services in China (OR = 0.29, 95% CI = 0.19–0.43, *p* < 0.001) than those from LICs.

**Table 3:** Adjusted odds ratios for participants’ health-related outcomes^a^

	Perceived good health status		Became healthier since coming to China		Ever had health problems in China		Had medical insurance in China		Perceived health services in China as good	
	OR_m_	(95% CI)	OR_m_	(95% CI)	OR_m_	(95% CI)	OR_m_	(95% CI)	OR_m_	(95% CI)
Countries by income levels										
LICs	1.00		1.00		1.00		1.00		1.00	
MICs	0.55	(0.29, 1.04)	0.81	(0.54, 1.22)	2.20	(1.25, 3.85)**	1.12	(0.75, 1.67)	0.83	(0.60, 1.16)
HICs	0.41	(0.21, 0.80)**	0.33	(0.19, 0.56)***	3.53	(1.93, 6.44)***	2.63	(1.67, 4.15)***	0.29	(0.19, 0.43)***
Socio-demographic characteristics										
Gender	–						–		–	
Male			1.00		1.00					
Female			0.52	(0.33, 0.81)**	1.44	(0.99, 2.09)^+^				
Age (years)	–								–	
18–25			1.00		1.00		1.00			
26–35			1.01	(0.64, 1.59)	0.89	(0.57, 1.40)	0.74	(0.50, 1.1)		
> 35			1.25	(0.72, 2.15)	0.67	(0.38, 1.20)	1.20	(0.74, 1.95)		
Education level	–		–		–				–	
Junior high or below							1.00			
Senior high school							1.74	(0.77, 3.89)		
College or above							2.75	(1.32, 5.72)**		
Current marital status	–								–	
Married			1.00		1.00		1.00			
Cohabitation			0.68	(0.33, 1.40)	1.95	(1.06, 3.61)*	2.57	(1.49, 4.43)**		
Single			0.76	(0.51, 1.14)	1.52	(0.99, 2.33)^+^	1.44	(1.00, 2.07)		
Migration-related characteristics										
Visa type	–		–						–	
Tourist visa					1.00		1.00			
Business visa					1.80	(0.98, 3.28)	1.16	(0.69, 1.94)		
Work visa					1.43	(0.80, 2.57)	2.37	(1.45, 3.88)**		
Student visa					1.26	(0.67, 2.38)	3.57	(2.06, 6.19)***		
Others					1.71	(0.86, 3.43)	1.97	(1.09, 3.56)*		
Number of visits to China	–		–				–			
Once					1.00				1.00	
2–5 times					1.26	(0.79, 2.02)			0.83	(0.60, 1.16)
6–10 times					1.40	(0.77, 2.55)			0.81	(0.52, 1.26)
> 10 times					1.65	(0.96, 2.81)			0.81	(0.56, 1.15)
Number of cumulative months of stay in China									–	
0–6	1.00		1.00		1.00		1.00			
7–12	1.12	(0.55, 2.30)	1.58	(0.96, 2.59)	1.21	(0.72, 2.03)	0.89	(0.56, 1.42)		
13–60	1.01	(0.56, 1.85)	1.21	(0.77, 1.91)	1.22	(0.76, 1.94)	1.41	(0.95, 2.09)		
61≤	0.45	(0.25, 0.79)**	1.02	(0.58, 1.77)	1.62	(0.95, 2.75)	1.07	(0.65, 1.75)		
Social integration in China										
Had a Chinese name	–									
No			1.00		1.00		1.00		1.00	
Yes			0.76	(0.54, 1.08)	1.15	(0.78, 1.70)	1.15	(0.82, 1.62)	0.83	(0.63, 1.11)
Had education experience in China	–		–		–				–	
No							1.00			
Yes							1.08	(0.74, 1.58)		
Proficiency in Mandarin	–		–							
Know nothing					1.00		1.00		1.00	
Know little					1.24	(0.72, 2.13)	0.94	(0.62, 1.42)	0.94	(0.66, 1.35)
Fair (can deal with daily life)					1.75	(0.96, 3.18)	0.91	(0.56, 1.48)	1.02	(0.67, 1.55)
Fluent					1.76	(0.88, 3.51)	0.66	(0.37, 1.17)	0.60	(0.36, 1.00)
Proficiency in English			–		–		–		–	
Know nothing	1.00									
Know little	2.48	(0.98, 6.26)								
Fair (can deal with daily life)	3.66	(1.51, 8.89)**								
Fluent	5.27	(2.22, 12.48)***								
Social network in China										
Number of Chinese acquaintances one knew in Guangzhou										
0–10	1.00		–		1.00		1.00		–	
11–50	1.70	(1.04, 2.78)*			1.04	(0.69, 1.56)	1.35	(0.96, 1.89)		
51≤	1.68	(1.03, 2.74)*			1.18	(0.78, 1.79)	1.32	(0.93, 1.88)		
Participation in hometown associations/the Chamber of Commerce in Guangzhou										
No	–		1.00		1.00		1.00		–	
Yes			1.68	(1.14, 2.49)**	1.24	(0.82, 1.89)	1.66	(1.14, 2.41)**		

^a^: Variables that were non-significant in univariate logistic regression models of all outcome variables were not listed in the table, namely currently has a job in China, number of years since the first visit to China, and number of fellow townsmen one knew in Guangzhou

--: Variables that were non-significant in univariate logistic regression models. ^+^: *p* < 0.1; ^*^: p < 0.05; ^**^: p < 0.01; ^***^: *p* < 0.001. Odds ratios (ORs) and 95% confidence intervals (CIs) with p < 0.05 are presented in bold

Having stayed in China for longer (OR = 0.45, 95% CI = 0.25–0.79, *p* < 0.01, Table 3) was independently and negatively associated with perceived good health status. Similar association was found between being female (OR = 0.52, 95% CI = 0.33–0.81, *p* < 0.01) and becoming healthier since arriving in China. Proficiency in English (OR = 3.66–5.27, *p* < 0.01) was positively associated with perceived good health status, and having a better social network in China (OR = 1.68–1.70, *p* < 0.05) was slightly positively associated with perceived good health status. Education (OR = 2.75, 95% CI = 1.32–5.72, *p* < 0.01), current marital status (OR = 2.57, 95% CI = 1.49–4.43, *p* < 0.01), visa type (OR = 1.97–3.57, *p* < 0.05) and social network in China (OR = 1.66, 95% CI = 1.14–2.41, *p* < 0.01) were also positively associated with having medical insurance in China.

## Discussion

This was the first quantitative study on the various characteristics and health-related perceptions of international migrants coming to China from HICs, MICs and LICs. The findings demonstrated that the majority of international migrants in Guangzhou came from MICs and LICs (75%). Although participants from the HIC group visited Guangzhou more frequently, those from the LIC group had longer stay. In addition, although a large proportion of the participants (89.6%) considered themselves to be in good health, one fifth of the participants had experienced health problems while staying in China and about half did not have health insurance. The results highlight potential gaps between the health care needs and the health care coverage of international migrants in China.

Migrants in China come from several countries. Over the 31 days of this studies, we recorded a very diverse group of migrants coming from 119 countries. Our finding highlighted the diversity of international migrants’ backgrounds in terms of countries of origin, languages, culture and religions. Such diverse backgrounds pose a challenge to the local health care system in coping with distinctive health needs and different expectations. The results of a US survey suggest that resources should be allocated to provide basic interpretation services to migrants in primary care and preventive health service centres [32], but such services are still absent in China.

About half of the participants had a Chinese name (47%) as many had an education experience in China (41.5%) or had an ongoing job in China (60.3%). Our findings also revealed that a significant proportion of migrants (about one third) came to China very frequently (more than 10 times). With such a high frequency of international mobility, the international migrants are more likely to be exposed to individual, social and environmental risks to health [33, 34], which may subsequently increase their vulnerability to health problems [35, 36]. Moreover, such high mobility could lead to potential risks of disease transmission. For example, both SARS and Zika viruses that recently caused outbreaks of contagious diseases in China and South America were originally carried by international migrants during their trips and led to transmission in local areas [37–39]. Therefore, it is important to strengthen the border surveillance and screening of infectious diseases among international migrants as well as multi-national cooperation on information exchange, joint investigation and health care policies to control pandemics [40].

Furthermore, one fifth of the participants reported to have experienced health issues while staying in China, while about half of the participants did not have medical insurance (especially those from LICs), and about 60% of the participants only knew little or no mandarin. This finding suggests the needs for offering effective medical services; for example, basic medical services with translators will be critical to meet the basic acute health care needs of international migrants, especially of those without health insurance. Reportedly, unfamiliarity with the health service process and lack of patient–physician language concordance are the primary difficulties faced by foreign residents seeking medical care in China [26]. Therefore, providing language assistance in clinics and leaflets with clear guidance and instructions of local health services (e.g. hospital address, standard procedure of seeing a doctor and average expenditure of seeing a doctor) in different languages may be helpful for foreign residents. For migrants with medical insurance, as the insurance itself does not ensure the use of health services, future studies investigating whether migrants who had medical insurance knew how to use it, whether they used it when in need, and what barriers they faced during the process are also warranted.

The findings further revealed that the income levels of the participants’ country of origin were independent factors affecting their health status, health insurance and perceptions of health services in China. Participants from HICs were more likely to have health problems, whereas those from LICs were more likely to report improved health status. Furthermore, 43% of the participants gave positive feedback regarding China’s health service. This percentage is lower than the percentages in a previous study on Chinese local residents, 76.5 and 67.2% of whom reported being satisfied with outpatient and inpatient services, respectively [41]. International migration status of the migrants contributes to the difficulties faced by them in seeking and using health services. Because of the limited health care resources and current intense patient–physician relationship in China [42], innovative measures will be required to provide effective health services to foreign residents; different community resources (e.g. community clinics, NGOs providing services for foreigners and volunteers) could be explored, and different services could be integrated. In the meantime, more targeted research is warranted to acquire a deeper understanding of this health-related issue.

### Limitations

This study had several limitations. First, the study included only those international migrants who visited the Guangzhou Municipal Exit-Entry Administration Office to resolve visa issues during the 1-month study period. Thus, migrants who did not need to change visa during that period or who used other methods to resolve visa issues were not included in the study. Second, because the study period (January) was right after the Christmas holidays, during the winter holidays for Chinese universities, and before the Chinese New Year, it is likely that many foreigners may have left China for their home countries; therefore, in other months of the year, there could be more international migrants. Thus, caution should be exercised when generalising the findings to the entire international migrant population of China. Third, although confidentiality and privacy were guaranteed during the survey process, reporting bias may still exist as all data were self-reported under the circumstances. Finally, the cross-sectional study design limited the causal inference, and the relationships between variables could only be explained as associations. Despite these limitations, our study provided the first “snapshot” of the health status and health care service perceptions of international migrants in China. It is important for China to understand the key health problems of different export countries to better cope with the diverse health needs of different migrants.

## Conclusions

Health equity and a universal health care coverage were hailed as the principles of the Chinese health system in the ‘Belt and Road High Level Meeting for Health Cooperation’ held in Beijing in 2017 [43]. To promote health equity, the health needs of international migrants should be considered for developing health policies and reforming the health care system given the large number of migrants. In the current context of globalisation and the continuing growth of China’s economy, more international migrants in China can be expected, and it will undoubtedly warrant a reform of the local health system to adapt to the multi-cultural reality. Such reform will indeed play a positive role in strengthening health diplomacy between China and other partner countries and expand China’s contribution to global health development [44, 45].

Therefore, our findings regarding the diverse backgrounds of international migrants in Guangzhou (Guangdong Province), including the 119 countries of origin, the languages they used, and the different socio-economic background, will be the beginning of the understanding of the demands on the health care system. Due to the ‘Belt and Road’ strategy and ‘South–South collaboration’, more international migrants in China are expected in future. More future studies should be conducted to explore the health issues faced by international migrants and to develop pilot innovative interventions to meet their health needs and the requirement for pandemic surveillance.
